# Exoenzyme Y Contributes to End-Organ Dysfunction Caused by *Pseudomonas aeruginosa* Pneumonia in Critically Ill Patients: An Exploratory Study

**DOI:** 10.3390/toxins12060369

**Published:** 2020-06-04

**Authors:** Brant M. Wagener, Naseem Anjum, Sarah C. Christiaans, Morgan E. Banks, Jordan C. Parker, Adam T. Threet, Rashidra R. Walker, Kayla D. Isbell, Stephen A. Moser, Troy Stevens, Mikhail F. Alexeyev, Jonathon P. Audia, Wito Richter, Kierra S. Hardy, Lina Abou Saleh, Charity Morgan, Jean-François Pittet

**Affiliations:** 1 Department of Anesthesiology and Perioperative Medicine, University of Alabama at Birmingham, Birmingham, AL 35294, USA; bwagener@uabmc.edu (B.M.W.); naseemanjum@uabmc.edu (N.A.); sarahc.ucsf@gmail.com (S.C.C.); morganbanks@uabmc.edu (M.E.B.); jordanparker@uabmc.edu (J.C.P.); athreet1@gmail.com (A.T.T.); rashidra.walker@gmail.com (R.R.W.); kaylaisbell@gmail.com (K.D.I.); 2 Center for Free Radical Biology, University of Alabama at Birmingham, Birmingham, AL 35294, USA; 3 Department of Pathology, University of Alabama at Birmingham, Birmingham, AL 35294, USA; smoser@uabmc.edu; 4 Center for Lung Biology, College of Medicine, University of South Alabama, Mobile, AL 36688, USA; tstevens@southalabama.edu (T.S.); malexeye@southalabama.edu (M.F.A.); jaudia@southalabama.edu (J.P.A.); ricther@southalabama.edu (W.R.); la1621@jagmail.southalabama.edu (L.A.S.); 5 Department of Physiology and Cell Biology, College of Medicine, University of South Alabama, Mobile, AL 36688, USA; 6 Department of Microbiology and Immunology, College of Medicine, University of South Alabama, Mobile, AL 36688, USA; ksh1004@jagmail.southalabama.edu; 7 Department of Biochemistry and Molecular Biology, College of Medicine, University of South Alabama, Mobile, AL 36688, USA; 8 Department of Biostatistics, University of Alabama at Birmingham, Birmingham, AL 35294, USA; cjmorgan@uab.edu; 9 Departments of Surgery and Cell, Developmental, and Integrative Biology, University of Alabama at Birmingham, Birmingham, AL 35294, USA; 10 Center for Lung Injury and Repair, University of Alabama at Birmingham, Birmingham, AL 35294, USA

**Keywords:** Type III secretion system, pneumonia, ExoU, tau, amyloid

## Abstract

*Pseudomonas aeruginosa* is an opportunistic pathogen that causes pneumonia in immunocompromised and intensive care unit (ICU) patients. During host infection, *P. aeruginosa* upregulates the type III secretion system (T3SS), which is used to intoxicate host cells with exoenzyme (Exo) virulence factors. Of the four known Exo virulence factors (U, S, T and Y), ExoU has been shown in prior studies to associate with high mortality rates. Preclinical studies have shown that ExoY is an important edema factor in lung infection caused by *P. aeruginosa*, although its importance in clinical isolates of *P. aeruginosa* is unknown. We hypothesized that expression of ExoY would be highly prevalent in clinical isolates and would significantly contribute to patient morbidity secondary to *P. aeruginosa* pneumonia. A single-center, prospective observational study was conducted at the University of Alabama at Birmingham Hospital. Mechanically ventilated ICU patients with a bronchoalveolar lavage fluid culture positive for *P. aeruginosa* were included. Enrolled patients were followed from ICU admission to discharge and clinical *P. aeruginosa* isolates were genotyped for the presence of exoenzyme genes. Ninety-nine patients were enrolled in the study. ExoY was present in 93% of *P. aeruginosa* clinical isolates. Moreover, ExoY alone (ExoY^+^/ExoU^−^) was present in 75% of *P. aeruginosa* isolates, compared to 2% ExoU alone (ExoY^−^/ExoU^+^). We found that bacteria isolated from human samples expressed active ExoY and ExoU, and the presence of ExoY in clinical isolates was associated with end-organ dysfunction. This is the first study we are aware of that demonstrates that ExoY is important in clinical outcomes secondary to nosocomial pneumonia.

## 1. Introduction

*Pseudomonas aeruginosa* is a gram-negative, opportunistic pathogen that is ubiquitous in nature and causes severe nosocomial infections in humans [1]. *P. aeruginosa* is listed by the World Health Organization as a member of the list of Critical Priority 1, highly antibiotic resistant ESKAPE pathogens [2,3]. *P. aeruginosa* is a frequent cause of bronchopneumonia in critically ill and immunocompromised patients. Furthermore, *P. aeruginosa* infection causes significant economic burden as pneumonia is a frequent cause of sepsis. *P. aeruginosa* is also associated with increased mortality in intensive care unit (ICU) patients due to ventilator-associated pneumonia (VAP) [4,5]. Finally; *P. aeruginosa* is often virulent and exhibits antimicrobial drug resistance [1].

Previous studies have attempted to underscore the bacterium’s pathogenicity that leads to lung injury, bacteremia, sepsis and increased end-organ injury and mortality [6,7]. During host infection, *P. aeruginosa* upregulates the Type III secretion system (T3SS) that is used to intoxicate host cells with exoenzyme (Exo) virulence factors. There are four known exoenzymes, named ExoY, ExoU, ExoS and ExoT, which assist *P. aeruginosa* in evading host defense systems and remodeling cellular lipids. More specifically, each of these enzymes produce a distinct injury in the host [7,8,9,10]; for example, ExoS and ExoT interact with Ras and RhoA GTPases, respectively, to modulate the cytoskeleton and endocytosis activities. ExoS and ExoT also cause ADP ribosylation. ExoU is a phospholipase A_2_ (PLA_2_) that disrupts the integrity of the lipid membrane and ExoY is a promiscuous nucleotidyl cyclase that breaks down microtubules. A hallmark of the exoenzymes is their requirement for host factors to mediate their activation upon injection into a host cell [11].

ExoY is present in ~90% of *P. aeruginosa* bacteria [12]. It was first described as a soluble adenylyl cyclase, generating cAMP within the cytosolic compartment. More recent studies have demonstrated that this exoenzyme can generate multiple cyclic nucleotides including, to date, cAMP, cGMP, cUMP and cCMP [13,14,15,16]. Importantly, ExoY affects the pulmonary endothelium by inducing inter-endothelial cell gap formation, resulting in edema [17,18,19,20]. Finally, recent studies indicate that ExoY is involved in the breakdown of peripheral microtubules and subsequent release of tau and amyloid β. These events lead to decreased endothelial cell barrier integrity and increased end-organ injury in both the pulmonary system and the brain [21,22,23].

Preclinical studies reveal that ExoU is associated with increased drug resistance and mortality, followed by ExoS and ExoT in a mouse model [24]. Furthermore, clinical studies have also established that lung infection caused by *P. aeruginosa* strains expressing ExoU are associated with greater severity of disease and higher mortality [25]. Although ExoU has been associated with severe disease and higher mortality rate compared to ExoS and ExoT, the role of ExoY is not well understood. In this study, we sought to understand whether ExoY contributes to end-organ dysfunction and mortality in critically ill patients who developed a pneumonia caused by ExoY-expressing *P. aeruginosa*. To this end, we collected *P. aeruginosa* clinical isolates from mechanically ventilated patients, genotyped the strains for exoenzyme expression, and compared exoenzyme expression with patients’ clinical parameters such as end-organ dysfunction, mortality rate and drug resistance.

## 2. Results

### 2.1. Patient Recruitment and Demographics

**Figure 1:**
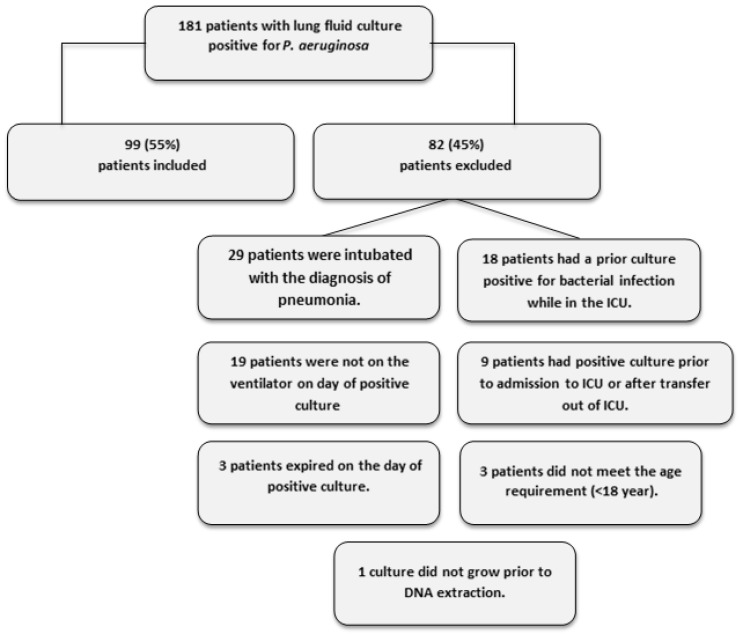
Flowchart of patient screening. Specific reasons for exclusion from the study can be found in the lower right hand side of the figure.

ExoU has been associated with increased mortality and drug resistance in previous studies [6,7]. To determine whether the presence of ExoY is associated with patient morbidity and mortality, we recruited patients with primary *P. aeruginosa* pneumonia to determine the prevalence of ExoY and correlate its presence with end-organ dysfunction. Over the 2-year study period, we screened 181 patients with primary *P. aeruginosa* lung infection (Figure 1). Of the 181 patients screened, 99 met inclusion criteria and were included in the study. Table 1 shows the demographics and characteristics of patients recruited to the study. Of note, the mean age was 57 years, males comprised 68% of the patients and 49% were admitted for acute trauma. Sixty-three percent were white, 33% were black and 6% were Hispanic. Social habits include that 33% consume alcohol regularly, 29% were smokers and 11% had documented illicit drug use. At ICU admission, the median Acute Physiology and Chronic Health Evaluation (APACHE) II score, Injury Severity Score (ISS), Sepsis-related Organ Failure Assessment (SOFA) and Organ Dysfunction and Infection System (ODIN) score were 16, 18, 7 and 1, respectively (Table 2). Patients were in the ICU for a median of 24 days, were mechanically ventilated for a median of 21 days and were mechanically ventilated for a median of 8 days before the onset of *P. aeruginosa* infection.

### 2.2. ExoY is More Prevalent than ExoU in Patients with P. aeruginosa Pneumonia

To determine the prevalence of T3SS exoenzymes in our clinical *P. aeruginosa* isolates, a genotype analysis was performed (Table 3). There were 20/99 patients whose *P. aeruginosa* isolates have the ExoU. The prevalence of ExoY was high, present in 92/99 patients. Interestingly, the prevalence of ExoY without ExoU (ExoY^+^/ExoU^−^), hereafter referred to as ‘ExoY only’, was also high in ICU patients compared to ExoU without ExoY (ExoY^−^/ExoU^+^), hereafter referred to as ‘ExoU only’; 74 versus two patients, respectively. Five patients had neither ExoU nor ExoY (ExoY^−^/ExoU^−^).

### 2.3. Clinical Isolates Exhibit ExoU PLA_2_ Enzyme Activity In Vitro and During the Infection of Cultured PMVECs

**Figure 2:**
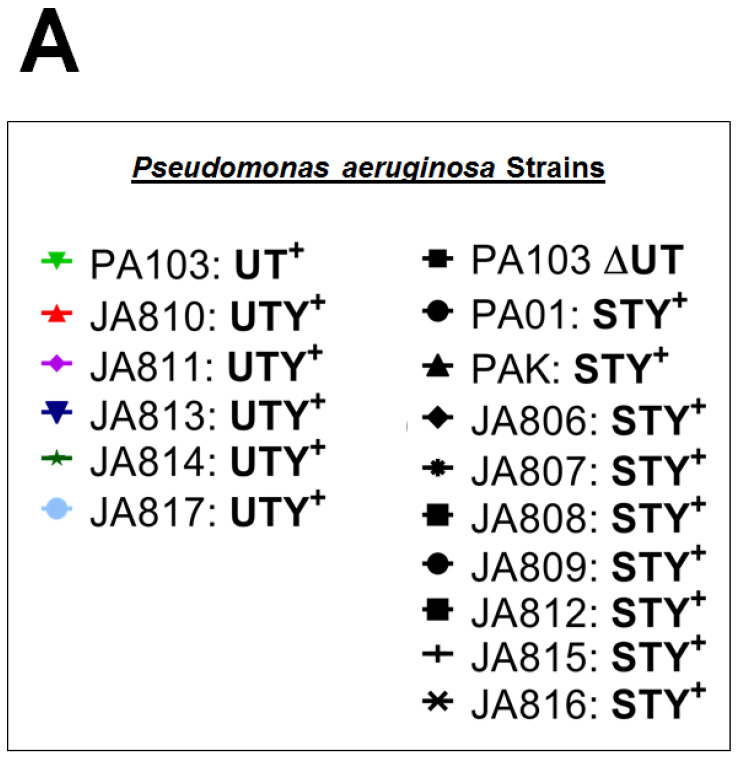

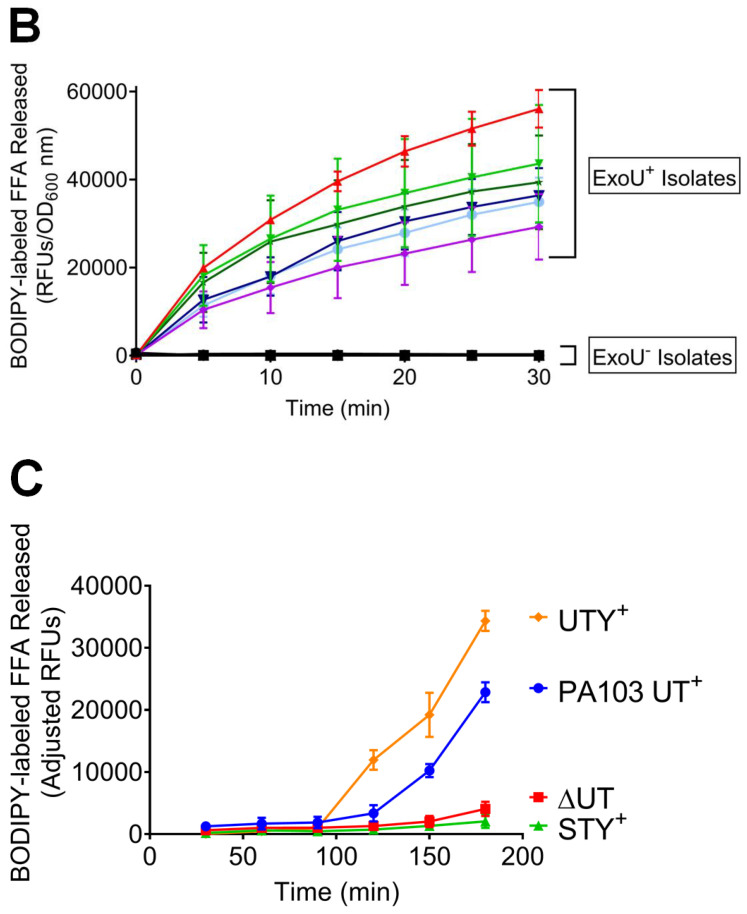
ExoU expression in bacterial isolates. (**A**) Bacterial cultures were selected randomly from patient isolates or lab strains known to possess ExoU per genotyping and grouped into ExoU^+^ or ExoU^−^ isolates for clarity. (**B**) Bacterial cultures were grown overnight. Single colonies were selected for inoculation into media with or without nitrilotriacetic acid (NTA) to induce the T3SS and secretion of exoenzymes to the culture medium. After overnight incubation, clarified supernatants were incubated with the fluorogenic PLA_2_ substrate PED6, and enzyme activity was measured with a spectrofluorometer. Data in the assay are expressed as relative fluorescent units (RFU) normalized to the OD600 of the overnight culture. Data at each time point are expressed as mean +/− SEM, run in triplicate, *p* < 0.05 comparing ExoU-positive isolates at 30 min to ExoU-negative isolates. (**C**) Confluent PMVECs were labeled with the PED6 substrate and subsequently exposed to bacteria with or without ExoU (at a MOI of 40:1). Supernatants were sampled over time to determine the presence of FFA using a spectrofluorometer. Data in the assay are expressed as relative fluorescent units (RFU), normalized to the OD600 of the overnight culture. Data at each time point are expressed as mean +/− SEM, run in triplicate, *p* < 0.05 comparing ExoU-positive isolates at 180 min to ExoU-negative isolates.

To determine whether clinical isolates from BAL fluid utilize ExoU and/or ExoY as part of their virulence arsenal, we performed functional studies assessing enzyme activity (Figure 2). Using an in vitro PLA_2_ activity assay, we showed significant enzymatic activity in all clinical strains which possess ExoU; however, variable activity among these strains was noted. PA103 is a reference lab strain known to encode a functional ExoU. This strain was initially isolated from a patient [26,27] and has been utilized extensively over the years as an ExoU^+^ strain. In Figure 2A, we have delineated which samples are ExoU-positive or -negative by listing the exoenzymes which are present. All strains that begin with “JA” are clinical isolates from our study, and all other strains are lab strains. In subsequent figures, we refer to these isolates as ExoU^+^ or ExoU^−^ for clarity. When compared to PA103, JA810 (one of the ExoU^+^ clinical strains isolated during our study) displayed higher PLA_2_ activity, whereas other clinical isolates had, to varying degrees, lower ExoU activity (Figure 2A). In contrast, none of the ExoU^−^ clinical isolates displayed phospholipase activity. As a negative control, we included a PA103 mutant devoid of ExoU (∆UT mutant). Thus, these data confirm our PCR genotyping assay and demonstrate that ExoU^+^ strains obtained from our patient cohort have PLA_2_ activity in vitro, whereas ExoU^−^ strains from these patients do not.

We next sought to confirm these in vitro phospholipase A_2_ (PLA_2_) assay results in an infection assay using cultured pulmonary microvascular endothelial cells (PMVEC) as a target host cell. We used PMVECs because pneumonia leads to increased alveolar-capillary permeability with exudative edema, commonly involving hemorrhage. In response to the complex inflammatory milieu generated during infection, capillary endothelial cells lose cell–matrix and cell–cell attachments, leading to a breach in barrier integrity. In some cases, cell retraction progresses to apoptosis and/or necrosis, which impair vascular recovery following infection. Bacteria virulence factors, including T3SS effectors, act on capillary endothelium and promote barrier disruption. Both PA103 and an ExoU^+^ clinical isolates generated the time-dependent accumulation of free fatty acid (FFA), indicating that ExoU was introduced from the bacteria into the host cell, where it activated phospholipase activity (Figure 2B). In contrast to ExoU-competent strains, the ∆UT mutant, in which exoenzymes T and U have been deleted from PA103, was without activity, and an ExoU^−^ clinical isolate displayed no activity. Overall, these findings support the assertion that clinical isolates possessing the ExoU gene have phospholipase A_2_ activity in an infection model. Similarly, ExoU deficient strains possess no phospholipase A_2_ activity.

### 2.4. Clinical Isolates Have ExoY Activity in PMVECs

**Figure 3:**
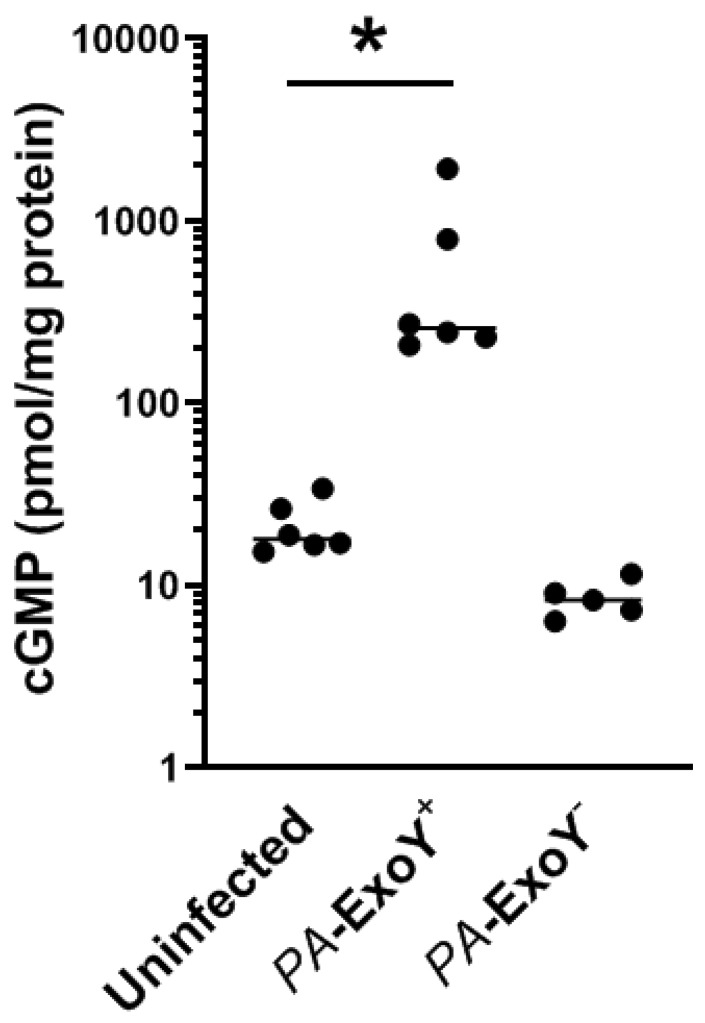
ExoY expression in bacterial isolates. Confluent layers of PMVECs were exposed for 6 h to distinct ExoY-positive (ExoY^+^/ExoU^−^) or ExoY-negative (ExoY^−^) strains at an MOI of 40:1, after which cells were harvested and intracellular cGMP levels were measured by enzyme immuno assay. Data are expressed as pmol cGMP per mg cell protein and are compared to uninfected PMVECs. * *p* < 0.05 when comparing *PA*-infected cells at 6 h to uninfected PMVEC.

We next examined ExoY activity in ‘ExoY only’ and ExoY-negative clinical isolates. ExoY is a promiscuous cyclase which generates both canonical (cAMP and cGMP) and non-canonical (cCMP and cUMP) cyclic nucleotides. Here, we measured cellular cGMP levels at 6 h post-infection, as cGMP was produced first and to the largest extent, after the infection with PMVECs infected with a lab strain expressing exogenous ExoY, as reported previously [14]. Our data indicate that ‘ExoY only’ clinical isolates can induce cGMP production in PMVECs (Figure 3). Compared to uninfected PMVECs or PMVECs infected with ExoY-negative clinical strains, they do so with highly variable activity, ranging from ~200 to 2000 pmol cGMP per mg protein in the six strains tested. These data illustrate that the enzyme entered the endothelium, acquiring its functional tertiary structure and interacting with its necessary co-factor, which is thought to be actin [17]. Thus, ExoY competent clinical strains utilize ExoY as a part of their virulence arsenal. They are able to introduce an enzyme that acquires activity in host cells.

### 2.5. End-Organ Injury Attributable to ExoY and ExoU

To better understand how ExoY may affect patient morbidity, we evaluated whether the presence of ExoY was associated with the development of acute kidney injury (AKI), cardiovascular dysfunction (CV), or coagulopathy (Table 4). Definitions of these measures of end-organ dysfunction can be found in the Methods section. We evaluated all patients in the study and then separately analyzed patients with isolates containing ExoY without ExoU (ExoY^+^/ExoU^−^) and patients with isolates positive for ExoU (with and without ExoY) (Table 4). Of note, data were not available for all patients or could not be measured for reasons listed in the Methods section. For AKI as a measure of end-organ dysfunction, 42/94 patients in the study were diagnosed with AKI in the week after a diagnosis of pneumonia. Of patients harboring ExoY only isolates, 35/71 had AKI and 6/18 patients harboring ExoU isolates had AKI. Regarding cardiovascular dysfunction, 42/99 patients in the study required vasopressors to support their blood pressure and maintain perfusion to critical end organs in the week after a diagnosis of pneumonia. For patients who were infected with ExoY only isolates, 28/74 required vasopressors, compared to 6/20 patients infected with ExoU isolates. Finally, 72/92 patients in the study had coagulopathy after their diagnosis of pneumonia. For patients with ExoY only isolates, 54/70 had coagulopathy compared to 14/17 patients with ExoU isolates. There is a clinical trend in the AKI group (but no statistical significance, *p* = 0.225) when comparing ExoY alone to ExoU towards more AKI when patients have isolates with ExoY only. Patients with ExoY only isolates had slightly more CV dysfunction compared to patients with clinical strains positive for ExoU, and patients with ExoY only isolates had slightly less coagulopathy compared to patients with isolates positive for ExoU. Importantly, the presence of ExoY alone is associated with end-organ dysfunction.

### 2.6. Mortality and Drug Resistance in ExoY^+^ Clinical Isolates

Pneumonia caused by ExoU expressing *P. aeruginosa* is known to be associated with illness severity, high mortality and antibiotic drug resistance [25]. However, the importance of the presence of ExoY by *P. aeruginosa* for illness severity and mortality remains unknown. Because only 2 patients in our cohort had pneumonia with ExoY^−^/ExoU^+^ *P. aeruginosa*, we could not statistically compare the mortality associated with the presence of ExoY without ExoU in our study with the mortality associated to ExoU. However, 17/74 patients with ExoY^+^/ExoU^−^ clinical strains died and 20/92 patients with any ExoY (ExoU-positive and –negative) died, with rates of 23% and 21.7%, respectively (Table 3). Furthermore, we identified clinical isolates with either single- or multi-drug resistance. In this study, 6/74 (8.1%) of patients with ExoY^+^/ExoU^−^ clinical strains and 6/92 (6.5%) of patients with any ExoY had single-drug resistance. Interestingly, multi-drug resistance increased for patients compared to single-drug resistance; 13/74 (17.6%) and 16/92 (17.4%) had multi-drug resistance in ExoY^+^/ExoU^−^ clinical strains and patients with any ExoY, respectively.

## 3. Discussion

*P. aeruginosa* is one of the major bacterial pathogens that causes acute pulmonary infections and is associated with significant morbidity and mortality in critically ill and immunocompromised patients [9]. Among the toxins produced by *P. aeruginosa*, preclinical studies have shown that T3SS exoenzymes play a major role in the virulence of that bacterium [6,7,8,9,10]. There is thus a growing interest in determining whether there is an association between the genetic presence of these T3SS exoenzymes and the outcome of critically ill patients who develop infection with *P. aeruginosa* [28,29,30]. Of the four exoenzymes, the presence of ExoU has previously been shown to be associated with drug resistance and increased mortality in patients with *P. aeruginosa* pneumonia [6,31].

ExoY is a promiscuous cyclic nucleotidyl cyclase capable of generating canonical (cAMP and cGMP) and non-canonical (cUMP and cCMP) cNMPs. The ability to generate each of these cNMPs differs among cell types, although at this point, the mechanisms responsible for discriminating which cNMPs are produced remain unknown [14]. Nonetheless, these cNMPs activate protein kinase A, resulting in phosphorylation of multiple (incompletely described) effectors, including tau. Tau hyperphosphorylation results in its dissociation from microtubules leading to tubule collapse, which in turn elicits cell rounding [32]. These cellular events strictly require ExoY enzymatic activity, i.e., the ability to produce cNMPs.

It is, however, unknown whether ExoY may also cause morbidity and mortality in critically ill patients who develop *P. aeruginosa* pneumonia [12]. To determine whether ExoY was associated with patient morbidity and mortality, we recruited ICU patients with primary *P. aeruginosa* pneumonia to determine the prevalence of ExoY and correlate the presence of ExoY with mortality, drug resistance and the development of end-organ dysfunction in these patients. The presence of ExoY without the presence of ExoU was detected in the vast majority of isolates. Overall, 75% of the isolates were ExoY^+^/ExoU^−^ and only 2% were ExoY^−^/ExoU^+^. The results of the present study demonstrate that patients with ExoY^+^/ExoU^−^ isolates have significant mortality (23%) and single- or multi-drug resistance (8.1% and 17.6%, respectively). There is no way to statistically compare these data to patients harboring ExoU^+^ clinical isolates because the numbers are too few in this study. Additionally, prior studies examining ExoU and mortality did not examine ExoY at the time of the study; therefore, reasonable statistical comparisons are not available. However, this is the first study to report mortality and drug resistance for patients with ExoY-containing isolates. Our study suggests that ExoY is likely a contributor to patient morbidity and mortality based on the percentage of patients that died due to infection with ExoY-positive isolates. 

In the last few decades, survival from critical illness (i.e., sepsis, septic shock, acute respiratory distress syndrome, etc.) has improved; however, in more recent years, the survival numbers have stabilized and no pharmacotherapy has been found to further improve survival [33,34,35,36,37]. Interestingly, while 28-day survival has improved for many patients, mortality at 1–2 years for these survivors is staggering [38,39,40]. Additionally, acute critical illness is now becoming chronic and patients are surviving with increased morbidity. Therefore, while measurement of mortality remains paramount, measurement of end-organ dysfunction is moving to the forefront as a mechanism of long-term morbidity. In our study, patients with ExoY-positive clinical isolates had coagulopathy and cardiovascular dysfunction, although it was not significantly different than ExoU^+^ isolates. There was a trend, though statistically insignificant, towards AKI. This is likely a Type II error, as more patients recruited to the study would have expanded this difference to statistical significance. How ExoY and/or ExoU contribute to these outcomes in end-organ dysfunction is currently unclear, but is the source of intense investigation.

Although mechanism(s) of end-organ dysfunction in survivors of critical illness remain unclear, recent preclinical studies demonstrate that pulmonary endothelial cell infection with *P. aeruginosa* causes extracellular release of oligomerized tau and amyloids in an ExoY- and ExoU-dependent manner [23,41]. First, oligomerized tau was released to the extracellular space after phosphorylation and degradation from microtubules. Furthermore, this tau was insoluble, could not be degraded with most techniques (resistance to enrichment with 50% ammonium sulfate precipitation and insensitivity to boiling, detergents, proteases, RNases and DNases) and was transmissible and propagated in naïve endothelial cells. Interestingly, tau was detected in the bronchoalveolar lavage fluid from patients with *P. aeruginosa* pneumonia and, when added to pulmonary endothelial cell monolayers, caused increased permeability in a dose-dependent fashion [41]. Additionally, amyloids were released after pulmonary endothelial cell infection with *P. aeruginosa*. Like tau, these amyloids demonstrated the ability to propagate in naïve endothelial cells and also displayed some insensitivity to degradation, indicating the behavior of a prion-like entity. In addition to being detected in the bronchoalveolar lavage fluid of patients with *P. aeruginosa*, they also disrupt long-term potentiation in the rat hippocampus [22,23]. Thus, generation of transmissible and cytotoxic amyloids within the lung may contribute to end-organ dysfunction that is due to ExoY and ExoU. Here, we report that patients infected with bacteria possessing ExoY were associated with significant cardiovascular events and coagulopathy; furthermore, we demonstrate a clinical trend towards more AKI and patients with ExoY clinical isolates. Future studies will address whether distinct amyloid species generated by ExoY and ExoU, respectively, account for this difference in end organ dysfunction.

This is the first study to report mortality and drug resistance for patients with ExoY-containing isolates, and the percentage of patients that died in this study with ExoY-positive isolates and pre-clinical studies indicate that ExoY is likely a contributor to patient morbidity and mortality. Limitations to our study include an inability to detect a difference in mortality between ExoY^+^/ExoU^−^ and ExoY^−^/ExoU^+^ isolates, given the discrepancy in the frequency of each within the study population. Furthermore, we have not screened ExoU activity or ExoY-dependent cyclic nucleotide levels in all 99 patient clinical isolates included in this study and hence cannot correlate the amount of exoenzymes produced in the patients with their health outcomes. However, detailed biochemical analysis of the strain-by-strain ExoU and ExoY activity is beyond the scope of the current manuscript.

In conclusion, ExoY is associated with patient mortality and end-organ dysfunction. In addition to ExoU, ExoY appears to be another virulence factor of *P. aeruginosa* pneumonia in critically ill patients. Future studies should further investigate the relative importance of ExoY protein expression as an important *P. aeruginosa* virulence factor and consider new treatments for inhibiting injection of T3SS toxins into parenchymal lung cells to prevent end-organ damage after *P. aeruginosa* bacteria are cleared from the lung by antibiotic therapy.

## 4. Conclusions

ExoY contributes to patient end-organ dysfunction. In addition to ExoU, ExoY appears to be another virulence factor of *P. aeruginosa* pneumonia in critically ill patients. Future studies should further investigate the relative importance of ExoY protein expression as an important *P. aeruginosa* virulence factor and consider new treatments for preventing the injection of T3SS toxins into parenchymal lung cells which continue to cause end-organ damage after *P. aeruginosa* bacteria are cleared from the lung by antibiotic therapy.

## 5. Materials and Methods

### 5.1. Study Design

Our study was a single-center, prospective observational study conducted at the University of Alabama at Birmingham (UAB) Hospital between November 2012 and January 2015. Patients were recruited from any of four intensive care units (Trauma–Burn Intensive Care Unit (ICU), Neuro ICU, Surgical ICU, or Medical ICU) at UAB hospital. All mechanically ventilated patients with a bronchoalveolar lavage (BAL) fluid culture positive for *P. aeruginosa* were considered if the infection was the patient’s first infection. Exclusion criteria included an age of less than 18 years, any prior positive culture from any source, pre-existing pneumonia on admission and/or prior to intubation, prisoners directly admitted from a correctional facility, and patients enrolled in an ongoing, interventional and randomized clinical trial. Patients were followed from ICU admission to ICU discharge and clinical data were prospectively collected on all patients enrolled in the study. For this study, we obtained an Exclusion of Informed Consent, as the bacterial isolates collected were considered ‘remnant tissue’ and no protected health information was obtained from the enrollees. This study was conducted in accordance with the Declaration of Helsinki and the protocol was approved by The University of Alabama at Birmingham Institutional Review Board (Protocol Number—X120513006, original approval date was July 19, 2012. The study remains under current approval).

### 5.2. Isolation and Identification of Clinical P. aeruginosa

BAL samples, submitted to the microbiology laboratory for bacterial culture, were vortexed for 30–60 s, followed by the inoculation of Trypticase Soy Agar II with 5% Sheep Blood, Chocolate Agar II and MacConkey Agar plates (Becton Dickinson, Sparks, MD, USA). Plates were then placed in an incubator containing approximately 5% CO_2_ at 35 °C for 24–48 h. Colonies from the quantitative primary media were counted, where each colony = 1000 colony-forming units (CFU) per milliliter [42]. Isolated colonies were identified by matrix assisted laser desorption ionization time of flight mass spectroscopy (MALDI-TOF, Vitek^®^MS, Biomerieux). Multiple stocks for each bacterial isolate were created by storing bacteria in 30% glycerol with media and keeping the stocks at −80 °C. These stocks were continuously used for this work; therefore, genetic drift due to continual bacterial growth is minimized.

### 5.3. PCR Method and Gel Electrophoresis

Clinical isolates of *P. aeruginosa* were genotyped by PCR using GoTaq Green 2× PCR master mix (Promega, Madison, WI, USA). Each 25 µL reaction contained 50–100 ng of *P. aeruginosa* genomic DNA and 0.5 µM concentration of each PCR primer. PCR primers were mixed into two groups, and forward and reverse sequences are listed in Table 5 (Mix1 = *exoU* + *exoT* + *polA*, Mix2 = *exoS* + *exoY* + *polA*). The *polA* gene was used as the internal control in all reactions. Initial denaturation was performed at 94 °C for 5 min, followed by 35 cycles of denaturation at 94 °C for 30 s, annealing at 55 °C for 20 s and extension at 72 °C for 50 s, followed by final extension at 72 °C for 3 min and holding at 4 °C. PCR products were run on 1% agarose gel, stained with ethidium bromide and imaged using a BioDoc-it system. The four exoenzymes genotyped were ExoS, ExoT, ExoU, and ExoY.

### 5.4. Measurement of Secreted ExoU Activity In Vitro

Multiple patient isolates that were ExoU^+^ or ExoU^−^ were randomly selected and screened for the presence of ExoU PLA_2_ enzymatic activity in order to validate the PCR results. We used a previously established assay by which bacteria grown in liquid culture can be triggered to produce and secrete T3SS effectors into the culture medium via the chelation of divalent cations [43]. To do this, bacterial cultures were grown from frozen stocks onto LB agar medium at 37 °C overnight. Single colonies were inoculated into Trypticase Soy Broth (TSB) liquid cultures supplemented with monosodium glutamate (0.1 M), glycerol (1%) and nitrilotriacetic acid (NTA, 0.01 M) (T3SS activation) or without NTA (no T3SS activation) and grown overnight with aeration at 32 °C. Under the +NTA conditions, exoenzymes are secreted directly into the culture medium. The optical density at 600 nm (OD600) was measured in the overnight liquid cultures for PLA_2_ assay normalization. Bacteria were removed from liquid overnight cultures by centrifugation and clarified supernatants (i.e., bacteria free) were used to measure ExoU PLA_2_ activity. PLA_2_ activity assays contained 14.5 µL of supernatant in a buffer solution containing 50 mM MOPS (pH 7.4) and 50 mM NaCl. To each reaction, we then added a fluorogenic PLA_2_ substrate, PED6 (N-((6-(2,4-Dinitrophenyl)amino)hexanoyl)-2-(4,4-Difluoro-5,7-Dimethyl-4-Bora-3a,4a-Diaza-s-Indacene-3-Pentanoyl)-1-Hexadecanoyl-sn-Glycero-3-Phosphoethanolamine, Triethylammonium Salt, 30 µM) followed by incubation at room temperature for 30 min. Subsequently, poly-Ubiquitin (0.1 mg/mL) was added to activate ExoU PLA_2_ activity [44] and samples were removed every 5 min. Negative control reactions did not have poly-Ubiquitin added. ExoU PLA_2_ activity results were generated by quantifying the release of BODIPY-labeled free fatty acid (FFA) from PED6 hydrolysis [45,46] using a nano-spectrofluorometer. Data are expressed as relative fluorescent units (RFU) normalized to the OD600 of the overnight culture.

### 5.5. Rat Pulmonary Microvascular Endothelial Cell Isolation and Culture

Rat pulmonary microvascular endothelial cells (PMVEC) were isolated as described [47]. Harvested cells were transferred to a growth medium and primary cultures were characterized by cobblestone morphology and endothelial cell markers. PMVECs were cultured in DMEM 10% FBS and 1% penicillin/streptomycin at 37 °C and 5% CO_2_.

### 5.6. Measurement of ExoU Activity in PMVECs

Bacterial cultures were again grown from frozen stocks onto minimal E salts agar medium at 37 °C overnight. They were scraped into 10 mL sterile normal saline, collected by centrifugation, resuspended into 1 mL of saline and the OD600 was then measured. Confluent PMVEC monolayers were seeded at confluence prior to bacterial inoculation as previously described [48]. PMVECs were washed with serum- and phenol red-free DMEM and incubated in DMEM containing PED6 (30 µM) fluorogenic substrate for 1 h at 37 °C, 5% CO_2_. After 1 h, media were removed from the cells and replaced with fresh DMEM-containing bacteria at a multiplicity of infection (MOI) of 40:1. Medium samples were removed from the culture at the times indicated and immediately read on the nano-spectrofluorometer to determine the amount of BODIPY-FFA released during infection. A control well of cells labeled with PED6, but without infection, was sampled over the same time course, and these values for each time point were subtracted from the corresponding infection (experimental) samples to normalize the data for the endogenous background release of BODIPY-FFA from the host cells. Data are expressed as normalized RFUs.

### 5.7. Measurement of ExoY Activity in PMVECs

Multiple patient isolates that were ExoY^+^/ExoU^−^ and ExoY^−^ were randomly selected for the measurement of their ExoY activity. PMVECs were seeded and grown to confluence in 6-well plates. Bacterial strains were prepared for infection, as previously described [32], and bacteria were incubated on PMVECs at a MOI of 40:1. After 6 h, cells were washed, collected and cGMP was measured as previously described [14]. 

### 5.8. Determination of End-Organ Dysfunction

All patients were examined for acute kidney injury (AKI), cardiovascular dysfunction and coagulopathy after their diagnosis of *P. aeruginosa* pneumonia. For AKI, we used the Kidney Disease Improving Global Outcomes (KDIGO) definition for creatinine only [49]. After a diagnosis of primary pneumonia, if the patient’s serum creatinine increased by 0.3 mg/dL over 48 h this was considered a positive diagnosis of AKI. Patients were evaluated in 48-h periods from the diagnosis of pneumonia for a total of 7 days. Patients were given a positive diagnosis of AKI if continuous renal replacement therapy was present in the week after the diagnosis of pneumonia. If a patient had a diagnosis of end-stage renal disease, they were excluded from this particular analysis. For CV dysfunction, we evaluated all patients for the presence of absence of a vasopressor in the week after their diagnosis of pneumonia. For coagulopathy, we evaluated patients’ international normalized ratio (INR) for a week after a diagnosis of pneumonia. If, at any time, the INR was ≥1.2, they were given a diagnosis of coagulopathy. Some patients did not have their INR analyzed during their ICU stay; these patients were excluded from this particular analysis.

### 5.9. Statistical Analysis

All demographic and clinical variables with continuous measures were expressed as means, and a 95% confidence interval and categorical variables were expressed as proportions. The Chi-square test was used to compare end-organ dysfunction with ExoY and ExoU in patients. For in vitro data, all normal data are mean ± SEM. For normally distributed data, one-way ANOVA followed by Dunnett’s test was used to compare three or more groups and Student’s t-test to compare 2 groups. Bonferroni correction was used to adjust multiple comparisons. Statistical analyses were done with Prizm GraphPad. A *p* value of ≤0.05 was considered statistically significant.

## Figures and Tables

**Table 1:** Patient demographics and characteristics. For each category, the number (n) and percentage (%) of patients (where applicable) in that category are provided.

Characteristics	n (%)
Total	99
Age in years	57
Gender (Male)	68 (68)
Admission Type	
Trauma	49 (49)
Other	50 (50)
Race	
White	63 (63)
Black	30 (30)
Hispanic	6 (6)
Alcohol Consumer	
Current	33 (33)
Former	3 (3)
Unknown	9 (9)
Smoking	
Current	29 (29)
Former	22 (22)
Unknown	9 (9)
Illicit Drug Use	
Current	11 (11)
Former	4 (4)
Unknown	12 (12)

**Table 2:** Patient clinical characteristics. For injury scores or measures of end-organ dysfunction, both the median and the range are provided. Definitions of acronyms can be found in the Results Section. Of note, the 4 Point Lung Injury Score was not available for two patients, and the Trauma ISS was only available for 27 patients.

Injury Scores	Median (Range)
APACHE II	16 (3–37)
SOFA	7 (0–16)
SAPS II	35 (13–67)
ODIN	1 (0–6)
4 Point Lung Injury	1.67 (0.33–3.33)
Trauma ISS	18 (4–45)
**Characteristics**	**Median (Range)**
Duration of Mechanical Ventilation, median	21 (3–118)
Length of ICU Stay	24 (3–118)
Time from Ventilation until Onset of pneumonia, median	8 (0–52)

**Table 3:** Relationship between presence of exoenzymes, mortality and drug resistance. For these data, isolates were grouped into the presence and/or absence of ExoY and ExoU. Both number (n) and percentage (%) of isolate groups that are drug resistant, died or survived the length of the study have been provided.

	Isolates (n)	Single-Drug Resistant (n, %)	Multi-Drug Resistant (n, %)	Died (n, %)	Survived (n, %)
ExoY^+^/ExoU^−^	74	6 (8.1)	13 (17.6)	17 (23)	57 (77)
ExoY^−^/ExoU^+^	2	0 (0)	0 (0)	0 (0)	2 (100)
ExoY^+^/ExoU^+^	18	0 (0)	3 (16.7)	3 (16.7)	15 (83.3)
ExoY^−^/ExoU^−^	5	2 (40)	1 (20)	2 (40)	3 (60)
All Isolates	99	8 (8.1)	17 (17)	22 (22)	77 (78)

**Table 4:** Relationship between end-organ dysfunction in all patients, patient with ExoY^+^/ExoU^−^ and patients with ExoU only. For AKI, five patients (three in the ExoY^+^/ExoU^−^ group) were excluded as they had a diagnosis of end-stage renal disease. For coagulopathy, seven patients (four in the ExoY^+^/ExoU^−^ group) did not have any data available during the timeframe examined.

	All Patients	ExoY^+^/ExoU^−^ Patients	All ExoU^+^ Patients
Acute Kidney Injury	42/94	35/71	6/18
CV dysfunction	42/99	28/74	6/20
Coagulopathy	72/92	54/70	14/17

**Table 5:** PCR sequences for exoenzyme genotyping. For each gene, the 5′ to 3′ sequence of the forward and reverse primers are listed. Expected band size for each PCR fragment generated is listed.

Primer Name	Primer Sequence	PCR Fragment Size
exoY forward	TGAGCGAGGACGGATTCTA	309 bp
exoY reverse	GATAGCCGTTGCCCTTGAT	
exoU forward	CTCAATGTACTCCCACGCATAG	406 bp
exoU reverse	CATCCTGGAATTCTGTCCACTC	
exoT forward	GCCGAGATCAAGCAGATGAT	405 bp
exoT reverse	GACAGGCTCGCCCTTTAC	
exoS forward	CATCAGGTAATGAGCGAGGTC	410 bp
exoS reverse	TTCAGGGAGGTGGAGAGATAG	
PolA forward	TTTCCTGCAGCCAGTTATCC	707 bp
PolA reverse	CAAGCTCAAGAGCACCTACA	
